# Unraveling sleep respiratory dysfunction in amyotrophic lateral sclerosis: Beyond the apnea-hypopnea index and sleep-related hypoxia

**DOI:** 10.1016/j.heliyon.2024.e32250

**Published:** 2024-05-31

**Authors:** Andi Nuredini, Dario Bottignole, Filippo Stragliati, Pietro Anceschi, Sonia Romano, Irene Pollara, Anna Abramo, Francesco Rausa, Liborio Parrino, Lucia Zinno, Carlotta Mutti

**Affiliations:** a Unit of Neurosciences, Department of Medicine and Surgery, University of Parma, Parma, Italy; b Sleep Disorders Center, Department of General and Specialized Medicine, University Hospital of Parma, Parma, Italy; c Neurology Unit, Department of General and Specialized Medicine, University Hospital of Parma, Parma, Italy

**Keywords:** Sleep recording, Amyotrophic lateral sclerosis, Diagnosis, Hypoventilation, Noninvasive ventilation

## Abstract

The timely introduction of non-invasive ventilation (NIV) is extremely relevant in the multidisciplinary management of patients affected by amyotrophic lateral sclerosis (ALS) and is based on the proper identification of red flags for early diaphragmatic exhaustion. Polygraphic sleep recording may provide insightful information on the ongoing respiratory impairment; in particular, atypical breathing patterns need to be recognized, as the application of current guidelines for sleep-related hypoxemia or sleep apnea may be insufficient for detecting early signs of diaphragmatic fatigue. We report the case of a 51-year-old man affected by ALS who was asymptomatic for breathing impairment, but whose nocturnal polysomnographic recording, despite not significant for obstructive sleep apnea nor for conventional hypoventilatory patterns, strongly suggested initial respiratory failure, as lately confirmed by the pulmonary follow-up. We discuss the advantages of including sleep recording in the clinical work-up of patients affected by ALS.

## 1 Introduction

Amyotrophic lateral sclerosis (ALS) is a progressive neurodegenerative disorder affecting upper and lower motor neurons in the brain, brainstem, and spinal cord. Respiratory muscle weakness is a major feature of the disease, and respiratory failure is the leading cause of death in ALS patients [1]. The early identification of diaphragm exhaustion is crucial in the diagnostic work-up of ALS, as it enables prompt intervention with non-invasive ventilatory (NIV) support to ameliorate the quality of life and prolong survival [2].

Respiratory dysfunction in ALS characteristically begins during sleep, favored by the physiological variation of breathing patterns throughout the night. Specifically, rapid-eye-movement (REM) sleep, with its associated muscle atonia involving the external intercostal and accessory respiratory muscles [3], can exacerbate the presence of subtle sleep-related breathing difficulties. With the progression of diaphragmatic weakness patients develop REM-related hypercapnia since the alveolar ventilation is too low to sufficiently exhale carbon dioxide. As the disease progresses, hypercapnia might spread to non-REM sleep stages, potentially leading to chronic hypercapnic respiratory failure with severe global sleep fragmentation. In the latest years, a progressive disorganization of sleep architecture in ALS patients has been demonstrated, irrespective of subjective perception. Sleep in ALS usually presents a drop in total sleep time, lower sleep efficiency, increased nocturnal awakenings, inverted stage 1 (N1)/stage 3 (N3) ratio, and reduced REM sleep. In parallel, as with other neurodegenerative disorders, a relevant decrease in sleep microstructure oscillations has been demonstrated [4]. Sleep can thus be deemed as a topical character in the disease development and, accordingly, its evaluation may support clinicians in the diagnostic assessment and follow-up of ALS patients. Unfortunately, patients with ALS rarely refer to sleep centers as part of their multidimensional evaluation, and so far, sleep recording is not included in the recommended work-up for breathing assessment in patients with this neuromuscular disorder.

We present the case of an Italian adult male patient affected by ALS with initial respiratory impairment, finally unraveled after the execution of a nocturnal cardio-respiratory recording.

## 2 Case report

DP is a 51-year-old man who was diagnosed with ALS in May 2019. He was a former smoker, and his personal history was significant for restless leg syndrome and a single episode of depression at the age of 39. He did not have previous pulmonary, thoracic, and otorhinolaryngologic disorders. His family history was unremarkable for neuromuscular or neurodegenerative diseases. Neurological symptoms appeared almost 29 months earlier when he started complaining about progressive weakness affecting exclusively his right arm for at least 12 months, leading to a diagnosis of ALS, with a flail arm phenotype [5]. In the following years, the disease generalized with subsequent fulfillment of the revised El Escorial Criteria for definite ALS. Genetic testing excluded mutations in a panel of 25 ALS-associated genes (including the most frequent *SOD1*, *TARDBP*, *FUS* mutations and the *C9ORF72* repeat expansion). At our evaluation in May 2022, he presented mild dysarthria and moderate dysphagia, tongue hypotrophy, weakness, and fasciculations. He did not present facial muscles weakness. Remarkable weakness of head flexors and extensors and marked hypotrophy and paralysis of upper limbs coexisted with only mild distal weakness of lower limbs. Bilateral ankle clonus and Babinski sign were present and widespread fasciculations involved the four limbs. The patient scored 27/48 on the revised ALS functional rating scale (ALSFRS-R). In particular, the maximum score was detected in the ALSFRS-R respiratory [[6], [7], [8]] and bulbar [[1], [2], [3]] subitems, indicating an optimal subjective perception of respiratory function. Specifically, he did not complain of any respiratory symptom, and his cough was valid (Peak Cough Flow 444.6 L/min, normal value > 270 l/min). Speech pathologist assigned a total score of 152/200 (cutoff value of 177 points) at the Mann Assessment of Swallowing Ability, indicating moderate dysphagia with mild aspiration risk and level 4 at the American Speech Language Hearing Association (ASHA)'s National Outcomes Measurement System scale, indicating safe swallowing with moderate cues to use compensatory strategies. Pharyngography confirmed a global slowdown and mild discoordination of the oral phase of swallowing, with distal transit preserved and no penetration in the airways. Oxygen pulse oximetry was 97 % in air, both in supine and standing body positions. Morning arterial blood gas (ABG) excluded hypercapnia (pH of 7.41, pO_2_ 110 mmHg, pCO_2_ 38.9 mmHg, HCO_3-_ 24.7). Pulmonary function tests (PFT) showed a reduced slow vital capacity (SVC), with similar percentages in the standing and supine position (respectively 52 % and 51 % of the predicted value). Diaphragmatic ultrasound showed normal excursion (18.7 mm at rest, 62 mm during forced inspiration, normal values 1–2 cm during tidal breathing, 6–10 cm during deep inspiration). Despite the absence of clear-cut symptoms suspected of respiratory impairment we completed the diagnostic work-up with a full-night cardio-respiratory recording.

**Fig. 1:**
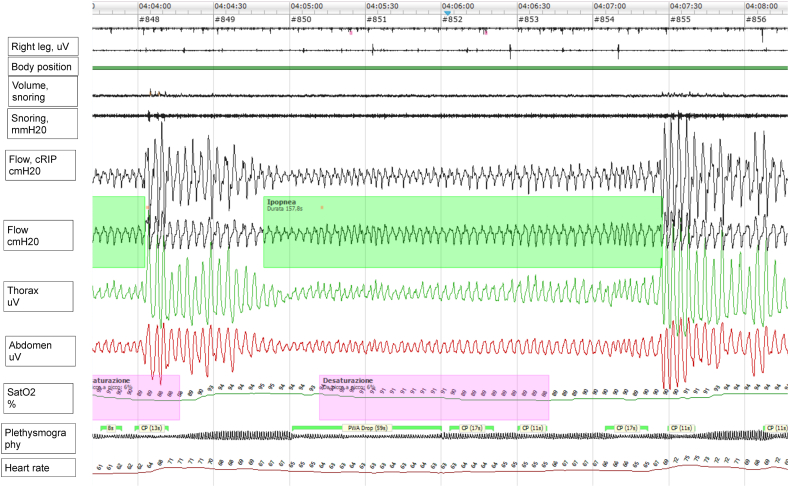
Example of a respiratory event in our patient, data collected from nocturnal cardio-respiratory recording (04 a.m.). Note the long-lasting hypopnea (157 sec in duration) characterized by irregular respiratory rate, highlighted in light green in the image, followed by a violent respiratory effort to restore normal breathing.

According to American Academy of Sleep Medicine (AASM) guidelines the exam documented an extremely mild form of obstructive sleep apnea (apnea/hypopnea index, AHI 5,8 events/h, ODI 3,6 events/h). He spent 2,4 % of the time below 90 %, thus not satisfying the criteria for sleep-related hypoxemia. However, upon visual inspection, we noticed the presence of extremely long-lasting obstructive respiratory hypopneas followed by abrupt and violent breathing restoration, highly indicative of diaphragm exhaustion. Events were collected in clusters, more prevalent in the second half of the night, and although we did perform complete PSG recording, the events were classified as suspected of REM-related respiratory hypopneas (Fig. 1). Clinically the patient complained about low sleep quality, with a Pittsburgh Sleep Quality Index score of 8, but with no daytime sleepiness (Epworth Sleepiness Scale of 7/24).

Despite the paucity of clinical symptoms and the reassuring power of some of the administered exams, which suggested a partially preserved pulmonary function, following the information provided by the cardio-respiratory monitoring, nocturnal NIV was introduced in June 2022, with clinical benefit. An overnight oximetry (ONO) repeated 4 months after NIV introduction excluded the progression towards a significant desaturation during sleep, with the time below 90 % of SpO_2_ being 0.9 %. A diaphragmatic ultrasound performed 10 months after NIV introduction showed values at lower limits of normal. We regularly check the efficacy and adherence to NIV via secure memory card. Parameters evaluated included: pressure (cmH20), leaks, percent days used, percentage days used >4 h, average use per night, residual AHI. The patient was adequately ventilated and, after the first period of adaptation to NIV, in few months he ameliorated his tolerance to the machine. At an 11-month follow-up the patient was still using NIV during the night and some hours in the afternoon, however, due to disease progression (sialorrhea, worsening secretions and major difficulties in mouth opening), tracheostomy was placed in June 2023.

## 3 Discussion

NIV is considered one of the milestones in the symptomatic treatment of ALS. By consenting the temporary resting of respiratory muscles affected by incipient denervation, NIV slows the decline of respiratory muscles strength, reduces the occurrence of acute adverse events, and prolongs survival in ALS patients [9,10]. ALS is a highly heterogeneous disease, with clinical phenotypes carrying distinctive clinical, demographic, and prognostic characteristics. Patients with flail arm phenotype have a relative benign course, with a median survival time of four years and up to 17.4 % of patients with a 10-years survival rate [11]. Remarkably, NIV introduction reduces motor decline as measured by the ALSFRS-R, in all disease stages, and independently from site of disease onset and disease severity, both in spinal and bulbar-onset phenotypes [12].

Albeit NIV benefits in patients affected by ALS have been largely confirmed, the timing for its introduction is still highly controversial. In the dynamic course of the disease, both clinical and instrumental features can support the identification of early signs of respiratory dysfunction. The 2010 National Institute for Health and Clinical Excellence UK (NICE) guidelines recommend starting NIV in asymptomatic patients if forced vital capacity (FVC) is less than 50 % of the predicted value or if SNIP and/or maximal inspiratory pressure (MIP) are less than 40 cmH_2_O, or if there is a decrease of SNIP/MIP of more than 10 cmH_2_O per 3 months [6]. The latest edition of EFNS Guidelines on Clinical Management of ALS suggests introducing NIV in the presence of respiratory symptoms and at least one abnormal respiratory function test, including a reduction of FVC to less than 80 % of the predicted value (while in previous guidelines the value was 50 %), SNIP lower than 40 cmH_2_O, PI max less than 60 mmH_2_O, a significant desaturation on ONO or an increase of more than 45 mmHg in morning blood gas pCO2 [7].

In clinical practice many indicators can be used to assess the severity of respiratory impairment including FVC/SVC, sniff nasal inspiratory pressure (SNIP) or cough peak flow. Nocturnal transcutaneous PCO_2_ (PtcCO_2_), although extremely useful, can be difficult to monitor and is not always available. Notably, conventional instrumental assessments can frequently lead to questionable results, and there is no consensus in clinical practice regarding the ideal timing for NIV initiation, making the decision to start or delay NIV really challenging at individual level. Lung spirometry in ALS patients with bulbar onset, may be misleading, due to high risk for air leaks and/or sub-maximal inhalation during the maneuver. In these cases, sleep recording can be even more important, to assess the real respiratory impairment of affected patients [8].

Furthermore, even in the presence of impaired exploratory tests, patients’ tolerance with NIV can sometimes delay its start. The role and impact of NIV acceptance in ALS have been rarely explored in literature. A retrospective study investigating 72 patients with ALS who started NIV in a multidisciplinary setting suggested that the presence of sialorrhea and neurobehavioral impairment, and the absence of respiratory symptoms are negative predictors of NIV adaptation [13]. At the time of NIV introduction, our patient presented mild sialorrhea and no subjective breathing disturbances making him hardly adapted to this approach.

In this complex framework, given that sleep represents the initial setting for breathing impairment evolution, its recording could make a significant difference, providing further elements useful to detect the very first signs of respiratory failure and/or to reinforce other instrumental findings when there is still uncertainty regarding NIV ideal initiation. Previous studies have warned about the limited utility of the ALSFRS-R respiratory subitems in predicting incipient respiratory failure in ALS [14]. Thus, objective tests are mandatory to ensure a reliable validation of patients’ condition. Literature evidence suggests that patients with various sorts of diaphragmatic weakness, including neuromuscular disorders, especially when symptomatic for non-refreshing sleep, should undergo sleep recording, to rule out the coexistence of obstructive sleep apnea (OSA) [15,16]. Although this first point is key in the timely identification of patients deserving further attention from sleep clinicians and pulmonologists, we emphasize the importance of other less common nocturnal breathing patterns that can be observed in polygraphic sleep recording, which could help predicting the ongoing respiratory failure. This could be particularly useful in patients affected by ALS, to select patients deserving non-invasive ventilatory support.

The American College of Chest Physicians Clinical Practice Guideline and Expert Panel Report endorsed by the American Academy of Sleep Medicine, the American Association for Respiratory Care, the American Thoracic Society, and the Canadian Thoracic Society suggest the utilization of polysomnography and/or overnight oximetry to rule out the presence of subclinical respiratory failure [17]. According to their recommendation, clinicians should look for either OSA or sleep-related hypoventilation/sleep-related hypoxemia in patients affected by neuromuscular disorders. Our patient did not present sleep apneas, or hypercapnia at wake, and he spent 2,4 % of the time below 90 % of SpO_2_. Hence, he did not fulfill the criteria for sleep-related hypoxemia, defined as oxygen saturation ≤88 % lasting for at least 5 min on nocturnal pulse oximetry according to the current ICSD-3 (2014) recommendation. However, at visual inspection, he presented clusters of long-lasting obstructive hypopneas, followed by violent and abrupt re-opening of the upper airways, highly suggestive of early signs of REM-related diaphragm exhaustion.

Common metrics for polygraphic sleep scoring can be misleading in faithfully representing diseases’ severity. In recent years, growing attention has been dedicated to the identification of factors determining the severity of OSA, moving beyond the (highly simplistic) AHI paradigm [18]. Indeed, AHI demonstrated to fail when it comes to a representative phenotypization of OSA patients [19]. Other factors, such as the hypoxic burden or novel measures for sleep-related respiratory events, should be adopted to capture the manyfold severity of the disease [20,21]. Similarly, we cannot claim to understand the complexity of the mechanisms involved in ALS-related respiratory failure using only the AHI or the time below 90 % of SpO_2_. The in-depth evaluation of polygraphic sleep recording can provide some essential details on nocturnal breathing patterns that sleep experts are aware of. Therefore, novel instrumental definitions for hypoventilation are desirable to allow a proper description of these patterns. In our patient we did not perform a video-PSG recording to confirm the REM-relatedness of respiratory events or to assess sleep architecture. Previous investigations documented that in ALS, even if patients subjectively perceived sleep as refreshing, sleep structure may be altered both at the macro and micro-structure level associated with the severity of underlying autonomic unbalance, with sleep-related predominance of sympathetic over parasympathetic tone [4,22]. While video-PSG may be complex and time-consuming to perform in each patient affected by ALS, cardio-respiratory polygraphic sleep recording is a repeatable, non-invasive, and inexpensive and highly informative exam. It is a multiparametric test inclusive of airflow signals, thorax and abdomen belts, microphone for snoring detection, plethysmogram, oxygen saturation signal, body position sensor, electrocardiogram (EKG) and electromyogram (EMG) for leg/arms movements. Although sleep polysomnography (comprehensive of scalp EEG channels), still represents the gold-standard to study sleep texture (inclusive of sleep macro and microstructure), current AASM guidelines recommend the utilization of cardio-respiratory recording for the diagnose of sleep-breathing disturbances [23].

We strongly believe that sleep recording might enrich the multidisciplinary evaluation of patients affected by ALS. Further studies involving larger samples and inclusive of the different ALS phenotypes are desirable to confirm our observations. We also suggest looking for subtle signs of diaphragmatic exhaustion, moving beyond the AHI metrics, to avoid the overlooking of prominent clues for hypoventilation which can help in the timely introduction of ventilatory support.

## Disclosure of conflicts of interest

The authors declare no financial or other conflicts of interest.

## Ethics statement

The case report has been performed following the ethical standards of the 1964 Declaration of Helsinki and later amendments. Informed written consent was obtained from the patient for the publication of this manuscript.

## Data availability statement

no data was used for the research described in the article.

## CRediT authorship contribution statement

**Andi Nuredini:** Writing – review & editing, Writing – original draft, Methodology, Investigation, Formal analysis, Data curation, Conceptualization. **Dario Bottignole:** Writing – review & editing, Formal analysis, Data curation. **Filippo Stragliati:** Writing – review & editing, Formal analysis, Data curation. **Pietro Anceschi:** Writing – review & editing, Investigation, Data curation. **Sonia Romano:** Writing – review & editing, Methodology, Data curation. **Irene Pollara:** Investigation. **Anna Abramo:** Investigation. **Francesco Rausa:** Writing – review & editing, Methodology, Investigation, Data curation. **Liborio Parrino:** Writing – review & editing, Visualization, Supervision. **Lucia Zinno:** Writing – review & editing, Supervision. **Carlotta Mutti:** Writing – review & editing, Writing – original draft, Supervision, Methodology, Investigation, Formal analysis, Data curation, Conceptualization.

## Declaration of competing interest

The authors declare that they have no known competing financial interests or personal relationships that could have appeared to influence the work reported in this paper.
